# NMR reveals a dynamic allosteric pathway in thrombin

**DOI:** 10.1038/srep39575

**Published:** 2017-01-06

**Authors:** Lindsey D. Handley, Brian Fuglestad, Kyle Stearns, Marco Tonelli, R. Bryn Fenwick, Phineus R. L. Markwick, Elizabeth A. Komives

**Affiliations:** 1 Department of Chemistry and Biochemistry, University of California, San Diego, 9500 Gilman Drive, La Jolla, CA 92093-0378, USA; 2 NMRFAM University of Wisconsin, 433 Babcock Drive, Madison, WI 53706, USA; 3 Department of Integrative Structural and Computational Biology and Skaggs Institute for Chemical Biology, The Scripps Research Institute, 10550 North Torrey Pines Road, La Jolla, CA 92037, USA; 4 San Diego Supercomputer Center, University of California, San Diego 10100 Hopkins Dr, La Jolla, CA 92093, USA

## Abstract

Although serine proteases are found ubiquitously in both eukaryotes and prokaryotes, and they comprise the largest of all of the peptidase families, their dynamic motions remain obscure. The backbone dynamics of the coagulation serine protease, apo-thrombin (S195M-thrombin), were compared to the substrate-bound form (PPACK-thrombin). *R*_*1*_, *R*_*2*_, ^15^N-{^1^H}NOEs, and relaxation dispersion NMR experiments were measured to capture motions across the ps to ms timescale. The ps-ns motions were not significantly altered upon substrate binding. The relaxation dispersion data revealed that apo-thrombin is highly dynamic, with μs-ms motions throughout the molecule. The region around the N-terminus of the heavy chain, the Na^+^-binding loop, and the 170 s loop, all of which are implicated in allosteric coupling between effector binding sites and the active site, were dynamic primarily in the apo-form. Most of the loops surrounding the active site become more ordered upon PPACK-binding, but residues in the N-terminal part of the heavy chain, the γ-loop, and anion-binding exosite 1, the main allosteric binding site, retain μs-ms motions. These residues form a dynamic allosteric pathway connecting the active site to the main allosteric site that remains in the substrate-bound form.

Serine proteases are found ubiquitously in both eukaryotes and prokaryotes, and they comprise the largest of all of the peptidase families with currently over 57,000 sequences in the MEROPS database and 3005 structures in the Protein Data Bank^1^. Their critical functions include intestinal digestion (trypsin and chymotrypsin), IgA-mediated immune response (tryptase, chymase, and the granzymes), control of dorsal-ventral signaling pathways of drosophila, blood coagulation, and complement activation. Serine proteases share a Ser, His, Asp catalytic triad and a greek-key, double β-barrel structure held together by disulfide bridges^2^. Sequence alignments with trypsin and chymotrypsin revealed that the coagulation proteases have non-conserved insertions at the base of several surface loops^3^. It has been suggested that these larger loops are responsible for the marked increase in substrate specificity^2^. In the case of the coagulation proteases, which cleave peptide bonds after arginine residues, the larger loops allow each coagulation protease to have stringent specificity so that they cleave only a few protein substrates.

The terminal protease in the coagulation cascade, human α-thrombin^4^,^5^, cleaves twelve known substrates causing fibrin clot formation, platelet activation, and thrombomodulin (TM)-mediated feedback inhibition of coagulation via inactivation of cofactors Va and VIIIa through activation of protein C. The allosteric regulation of thrombin specificity by its many effector molecules and towards its many substrates is not yet fully understood^6^,^7^. We recently completed computational studies which revealed temporal fluctuations of the apo-thrombin ground-state that are largely uncorrelated^8^. Binding of TM to the anion-binding exosite 1 (ABE1) of thrombin increased the collective correlated motions and this effect extended from the TM binding site into the active site loops, a result that was also seen upon active site occupation^8^,^9^. These computational studies suggest that effector and/or substrate binding alters the conformational ensemble of thrombin to affect distal functional regions. However, NMR experiments, which are well-suited to directly reveal such re-distributions of states^10^, have been slow to emerge due to the difficulties of preparing thrombin samples for NMR study^11^,^12^.

Many x-ray crystallographic structures of thrombin are available and these reveal thrombin surface loops “trapped” in different conformations^13^. However, the core structure of thrombin with different effectors and/or with or without bound substrate, do not reveal significant conformational changes. On the other hand, hydrogen-deuterium exchange followed by mass spectrometry showed rapid exchange of many of the backbone amides in thrombin, particularly in the surface loops^14^,^15^ and NMR experiments showed missing resonances^11^,^12^ both suggesting that thrombin may be dynamic.

We recently characterized the dynamics of active site-liganded thrombin (D-Phe-Pro-Arg chloromethylketone (PPACK)-thrombin) using a combination of NMR experiments and accelerated molecular dynamics (aMD) simulations. Dynamics on the ps-ns time scale were determined from *R*_1_, and *R*_2_ relaxation rates and ^15^N-{^1^H}NOE measurements analyzed by the Model-free approach. Measurements of *R*_ex_ using the TROSY-Hahn-Echo experiment revealed structural fluctuations on the μs to ms timescales^12^. Conventional MD simulations accurately recapitulated the ps-ns motions, and aMD simulations accurately recapitulated the broader motional ensemble captured in residual dipolar couplings. The aMD results revealed temporal fluctuations of the protein in the ground-state basin resulting in a broad structural ensemble with several highly dynamic surface loops, even in the active-site liganded form.

NMR has become the preferred experimental approach for investigating allosteric networks in enzymes. Allostery is often revealed by relaxation dispersion measurements which probe changes in μs-ms motions upon binding of substrates or different ligands^16^,^17^,^18^,^19^,^20^,^21^. Here we present the complete experimental NMR backbone dynamics profile of apo-thrombin as mimicked by the S195M active site mutant compared to that of thrombin covalently bound to the substrate analog, PPACK. The combined results for fast and slow timescales demonstrate that much of apo-thrombin has motions on the μs-ms time scale. Interestingly however, the central region of the light chain does not display ^15^N relaxation dispersion on the μs-ms timescale or low ps-ns order parameters, suggesting that the light chain provides an architectural clamp between the two beta barrels in the serine protease. The measured μs-ms motions of apo-thrombin reveal substantial motions of surface loops and strands connecting them. As was seen for several other enzymes, substrate binding substantially dampens protein dynamics^22^,^23^. Remarkably, even in the PPACK-bound form, a pathway of residues that retain μs-ms motions extends from the ABE1 allosteric binding site to the active site revealing how ABE1 and the active site are allosterically coupled through dynamics.

## Results

### Resonance assignments and chemical shift perturbations

Because wild type apo-thrombin undergoes autocatalytic proteolysis, the conservative, yet catalytically dead S195M mutant was studied (hereafter referred to as apo-thrombin). As with PPACK-thrombin studied previously^12^, some backbone amides did not show peaks in the ^1^H-^15^N TROSY HSQC spectrum, probably due to line broadening resulting from conformational flexibility in the intermediate time scale. All observed resonances were assigned, accounting for 76% of the backbone amides in apo-thrombin. In comparison, the previously published assignments of the S195A mutant accounted for 68% of possible backbone amide resonances^11^.

**Figure 1:**
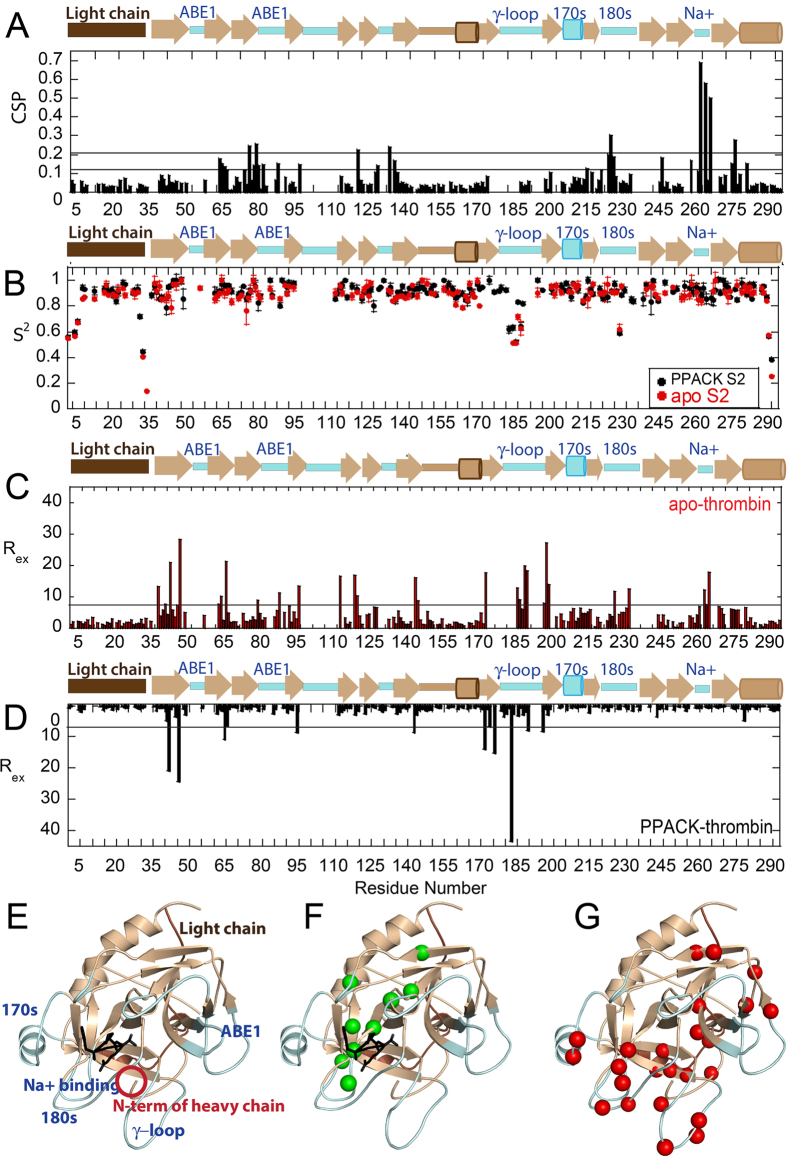
(**A**) A plot showing average weighted chemical shift differences between S195M-thrombin and PPACK-thrombin. Horizontal lines indicate 1 and 2 standard deviations above the mean. A schematic above the plot shows the surface loops colored in pale cyan. (**B**) The order parameters for PPACK-thrombin (black) and apo-thrombin (red) determined from *R*_1_, *R*_2_, and ^15^N-{^1^H}NOE measurements analyzed by the Model-free approach implemented in TENSOR2. (**C**) Plot showing *R*_ex_ for residues in apo-thrombin: 24 residues showed significant relaxation dispersion curves with an *R*_ex_ value > 6 Hz (horizontal bar). (**D**) Plot showing *R*_ex_ for residues in PPACK-thrombin: only nine of which showed an *R*_ex_ value > 6 Hz. This plot is reversed for ease of comparison with the plot in panel (**C**). (**E**) The structure of thrombin (1PPB) is shown with the light chain colored in brown, the backbone in sand and the loops in pale cyan. The relevant loops are labeled both in the structure and above the secondary structure elements in panels (**A**–**C**). PPACK is shown in black sticks. (**F**) The residues with chemical shift differences greater than 2 standard deviations are highlighted in green spheres on the structure of thrombin (1PPB) in the same orientation as in (**E**). (**G**) The residues with R_ex_ > 6 Hz in apo-thrombin are highlighted in red spheres are shown on the structure of thrombin depicted in the same manner as in panel E.

Forty residues for which N-H peaks were visible in PPACK-bound thrombin were not observed for apo-thrombin. These included five residues in the 90 s loop, nine in the γ-loop, two around the 170 s loop, five in the 180 s loop, a residue next to the catalytic serine, and three in the Na^+^-binding loop (Fig. 1E; note: To accommodate readers who use one of several different numbering schemes for thrombin, we report residues in the chymotrypsin numbering scheme in which loops are denoted 60 A, 60B, etc, followed by the sequential numbering used in the NMR data plots (given in parentheses)). The loops are also labeled according to the chymotrypsin numbering so that, for example, the 180 s loop corresponds to residues 225–238 in sequential numbering.). These residues were mostly found in the functional loops surrounding the active site suggesting that PPACK ligation reduces both the conformational and temporal range of dynamic motions, as suggested previously^11^. A large number of residues showed significant chemical shift differences between apo-thrombin and PPACK-thrombin. Residues with chemical shift differences twice the standard deviation (Δδ > 0.21 ppm, Fig. 1A) corresponded to almost the entire substrate specificity pocket residues G184(225), S214(262), G216(264), G219(266), and Y228(276) but also included distal residues T54(76), H57(79), Y89(121) and R101(134) (Fig. 1F). The chemical shift changes extend to parts of thrombin distal to the substrate binding site consistent with allosteric coupling throughout thrombin from the active site to effector binding sites^24^,^25^,^26^.

### Picosecond-nanosecond motions in thrombin

Amide bond vector order parameters (S^2^) calculated from Lipari-Szabo model-free analysis^27^,^28^ of *R*_1_ and *R*_2_ relaxation rates and ^15^N-{^1^H}NOEs measured for [U-^2^H], [U-^15^N]-labeled apo-thrombin reflect ps-ns motions. The majority of the residues in apo-thrombin could be fit to model 1 (58%) or model 2 (69%, with Models 1 and 2 combined). Few differences were observed in the ps-ns motions between apo and PPACK-thrombin (Fig. 1B) and those differences were accurately predicted by classical MD simulations^12^.

### Microsecond-millisecond motions in thrombin

Exploratory ^15^N-TROSY Hahn-Echo NMR experiments^29^ suggested that many more residues throughout apo-thrombin may be undergoing motions on the μs-ms time scale, than had been previously found in PPACK-thrombin^12^. Therefore, relaxation dispersion (CPMG) experiments were performed on both apo-thrombin and PPACK-thrombin at 600 MHz and 800 MHz^16^. To initially evaluate the μs-ms dynamics, we calculated the *R*_ex_ for each observed resonance from the CPMG data [*R*_ex_ = *R*_2_^obs^(1/τ_CPMG_^0Hz^) − *R*_2_^obs^(1/τ_CPMG_^max^)]. Using a cut-off of *R*_ex_ > twice the maximal duplicate error, 24 residues located throughout the apo-thrombin structure, with seven found in the N-terminal β-barrel and 17 in the C-terminal β-barrel, had μs-ms motions (Fig. 1C). PPACK-binding did not induce chemical shift changes in residues that were dynamic on the μs-ms time scale suggesting that the “excited states” that were being sampled did not simply correspond to the substrate-bound state (Fig. 1G – red spheres). All 24 relaxation dispersion curves for apo thrombin could be globally fit using GLOVE^30^ yielding the following parameters: *k*_ex_ = 1770 s^−1^ ± 60 s^−1^, p_b_ = 4.1% ± 0.6% (Supplementary Fig. 1). We note that *k*_ex_ is the sum of the forward and reverse rates for the conformational transitions, and given the low population of the excited state, the rate is dominated by the transition rate from excited state to ground state, and the transition rate from ground to excited state is at least 10-fold slower. Thus, a *k*_ex_ of 1770 s^−1^, represents a transition time from excited state to ground state of ~600 μs and from ground state to excited state of ~6 ms. PPACK-binding reduced the number of residues with *R*_ex_ > 6 Hz from 24 in apo-thrombin to nine (Supplementary Fig. 1).

### The stabilizing role of the light chain of thrombin

**Figure 2:**
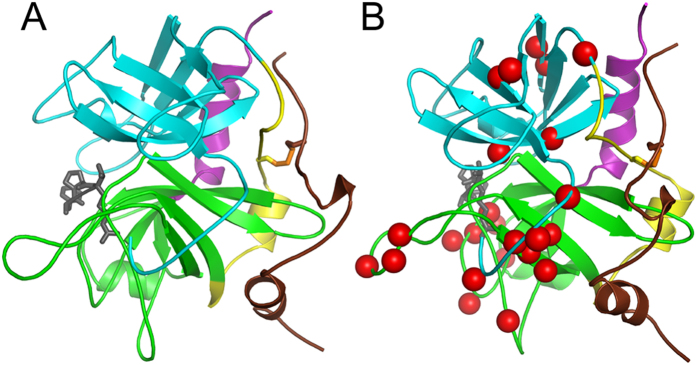
(**A**) Structure of thrombin showing the N-terminal β-barrel in cyan, the C-terminal β-barrel in green, the strand that links the two β-barrels in yellow, the C-terminal helix in magenta, and the light chain in brown. The PPACK is shown in grey sticks to mark the active site. The orientation was chosen to show the crosswise disposition of the light chain and the linking strand. (**B**) The molecule from panel A rotated ~90° and the residues undergoing μs-ms motions in apo-thrombin are marked with red spheres.

Although data were obtained for all but six residues within the light chain of apo-thrombin, the relaxation dispersion curves for every residue within the light chain was flat indicating the light chain is not dynamic on the μs-ms time scale. The lack of light chain motion was surprising because it is completely surface exposed and has relatively little regular secondary structure. Architecturally, the light chain is disulfide-bonded to the strand that links the N-terminal β-barrel to the C-terminal β-barrel, which also did not have any residues undergoing μs-ms motions (Fig. 2A). In fact, the entire surface of the thrombin molecule that contacts the light chain or the linker between the two barrels is completely devoid of μs-ms motions (Fig. 2B). This includes the C-terminal helix of thrombin. These results suggest that the light chain and the strand that connects the β-barrels form a clamp that holds the two β-barrels together. It is possible that increased entropy in the active site loops could compensate for the loss of entropy caused by the light chain clamp or that the rigidity of the light chain clamp allows for more extensive dynamics in at the active site loops.

### PPACK-binding quenches the dynamics of thrombin active site loops

PPACK-binding largely quenched the μs-ms motions in the loops surrounding the active site, whereas the ps-ns motions were largely unchanged (Fig. 1B)^12^. Similar observations have been made for several other enzymes^10^,^17^,^18^,^22^,^31^,^32^. Residues T172(213) and I174(215) in the 170 s loop/helix, R187(233) in the 180 s loop, and G216(264), E217(265), G219(266) and D222(270) in the Na^+^-binding/specificity loop had strong relaxation dispersion curves in apo thrombin, but showed no relaxation dispersion in the PPACK-bound state (Supplementary Fig. 1). These regions are all interesting for different reasons. The 170 s loop, which is highly frustrated^9^, may be exchanging between an inactive conformation and an active conformation. Substrate-binding (mimicked by PPACK) may select the more stable, proteolytically active conformation. Indeed, stabilization of the 170 s loop was shown to enhance activity in Factor VIIa^33^. Motions on the μs-ms timescale in the Na^+^-binding/substrate specificity loop were also completely dampened upon PPACK-binding, which may reflect the coupling between substrate binding and allosteric regulation by Na^+^ ions^34^,^35^.

### Motions within the N-terminus of the thrombin heavy chain

**Figure 3:**
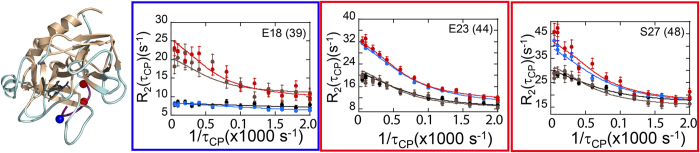
Relaxation dispersion curves for residues in the N-terminus of the thrombin heavy chain. Dispersion curves and data points for apo-thrombin at 800 MHz are depicted in red and 600 MHz in brown; those for PPACK-thrombin at 800 MHz are in blue and 600 MHz are in black. The dynamics of E18(39), closest to the N-terminus (blue sphere), were quenched by PPACK-binding. The dynamics of E23(44) and S27(48) were retained after PPACK-binding (red spheres).

The most N-terminal residue in the heavy chain for which we were able to obtain relaxation dispersion data was E18(39). This residue was highly dynamic in apo-thrombin, and its dynamics were entirely quenched upon PPACK binding. This result suggests that substrate binding stabilizes the N-terminal Ile-NH_3_^+^ that is inserted into “Ile cleft”. Interestingly, the dynamics of the neighboring residues, E23(44) and S27(48), do not change with PPACK binding. These residues contact the γ-loop, which is also very dynamic (Fig. 3). Recent HDXMS data showed a dramatic decrease in amide exchange in the N-terminus of the heavy chain upon PPACK-binding, however, some amides still exchanged within residues 16–33(37–44), indicating that part of this region is still exposed and/or dynamic^15^. The NMR results presented here now allow us to localize the change in dynamics to the most N-terminal residues 16–18 (37–39) of the thrombin heavy chain and to conclude that residues subsequent to these remain dynamic.

### Motions within the γ-loop of thrombin

**Figure 4:**
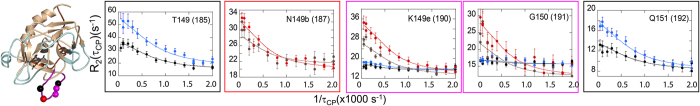
Relaxation dispersion curves for residues in the γ-loop of thrombin. The color scheme for the dispersion data is as in Fig. 3. T149(185) and Q151(192) had strong relaxation dispersion curves in PPACK-thrombin and were not observed in apo-thrombin (black spheres and plot border). N149b(187) was only observed in apo-thrombin (red sphere and plot border). K149e(190) and G150(191) were observed in both PPACK- and apo-thrombin and their dynamics were quenched by PPACK-binding (magenta spheres and plot borders).

Previously published protease sensitivity and amide exchange studies consistently showed that the γ-loop is much more dynamic in apo-thrombin than in PPACK thrombin^15^,^36^. Therefore it was not surprising that most of the NH cross peaks for γ-loop residues were missing, cross peaks for residues V138(174) in the β-strand leading into the γ-loop, and V158(199) and N159(200) in the β-strand exiting the γ-loop showed μs-ms motions in apo-thrombin (Supplementary Fig. 1). Cross peaks and strong relaxation dispersion curves were observed for residues T149(185) in the strand leading into the γ-loop, and Q151(192) in the strand exiting the γ-loop in PPACK-thrombin, but signals were missing altogether for these residues in apo-thrombin (Fig. 4). Residue N149b(187) at the tip of the γ-loop showed μs-ms motions in apo-thrombin. Residues K149e(190) and G150(191) showed μs-ms motions in apo-thrombin which were completely quenched upon PPACK-binding (Fig. 4). Taken together, these results suggest that the γ-loop is entirely more dynamic in apo-thrombin, with many of the cross peaks broadened beyond detection as a result of chemical exchange. PPACK-binding dampens the dynamics throughout the loop so that several residues that were missing in apo-thrombin show relaxation dispersion in PPACK-thrombin and motions of residues for which relaxation dispersion is observed in apo-thrombin are quenched in PPACK-thrombin.

### PPACK-thrombin retains μs-ms motions marking an allosteric pathway from ABE1 to the active site

**Figure 5:**
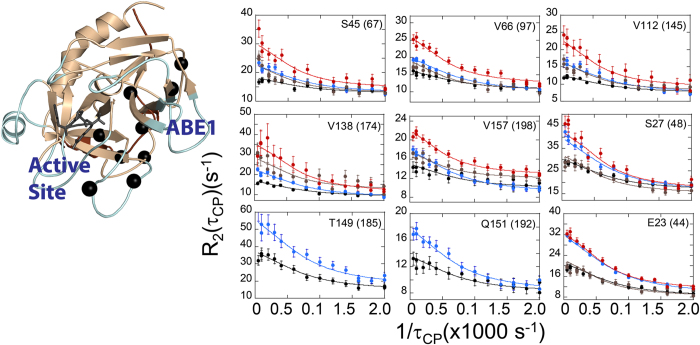
Relaxation dispersion curves for the nine residues that retained significant μs-ms motions in PPACK-thrombin. The color scheme for the dispersion data is as in Fig. 3. The plots are aligned with the residue location in the structure and corresponding residues are marked with black spheres.

Although PPACK-binding quenched most of the μs-ms motions in apo-thrombin, nine residues in PPACK-thrombin had significant relaxation dispersion curves. GLOVE fitting of these nine residues indicated fast timescale motions and therefore the exchange rate was determined by global fitting to the Meiboom equation^37^ yielding *k*_ex_ = 2440 s^−1^ ± 50 s^−1^. Five of the nine residues that retained μs-ms motions in PPACK-thrombin were in the N-terminal β-barrel and four were in the C-terminal β-barrel. The N-H peaks for two residues, Thr149(185) and Gln150(191), both in the γ-loop, were only visible in PPACK thrombin. The other seven residues showed μs-ms motions in both apo-thrombin and PPACK-thrombin. Remarkably, when mapped onto the structure of thrombin, all nine residues formed a pathway of residues moving on the μs-ms timescale from the ABE1 allosteric site to the active site (Fig. 5). Given that apo-thrombin shows μs-ms motions throughout the structure, these results suggest that apo-thrombin comprises a broad ensemble of conformations and that PPACK-binding selects for a subpopulation of this conformational ensemble that retains μs-ms motions only along this main allosteric pathway. Thus, as has now been reported for several allosteric proteins reviewed in^21^, the motions correlated with function and allostery in thrombin occur in the μs-ms time regime. The pathway of residues retaining μs-ms motions in the substrate-bound form extends through and includes both β-barrels in the thrombin structure. Previous aMD studies revealed correlated motions only in TM456-thrombin, but absent in apo-thrombin and thrombin-TM56. Remarkably, motions of the γ-loop were correlated with motions in EGF4 of TM456^8^. These results together suggest that the γ-loop mediates signals back and forth between the active site and ABE1.

## Discussion

While previous studies have mapped *R*_ex_^12^ and line broadening^11^, this study represents the first complete experimental characterization of motions from ps to ms in a serine protease. Apo-thrombin has a very large number of backbone N-H groups showing μs-ms motions (>9% of the total non-proline residues), consistent with previous aMD simulations which also showed significant motions up to the μs timescale throughout thrombin^8^. Residues throughout the core of the serine protease show μs-ms timescale motions. It is tempting to speculate that such internal motions may be due to the disulfide bonds sampling rotational conformers in the μs-ms time scale^38^. In addition, the disulfide bonds may stabilize a more loosely-packed protein core than is required for intracellular proteins, which must be stabilized by packing of secondary structural elements. The network of residues in thrombin moving on the μs-ms timescale also appears much larger than has been observed in other enzymes, which typically have a single loop that closes over the active site or two subdomains that close with respect to each other^22^,^23^. While others have been able to correlate allosteric networks with chemical shift differences between excited and ground states^39^, we did not observe a significant correlation between the chemical shift differences between apo and PPACK thrombin and those observed in the relaxation dispersion measurements. These results suggest that the PPACK-bound form does not correspond to the excited state we are observing in apo thrombin, a result that is not too surprising considering the number of different dynamic states that thrombin likely adopts during its complex catalytic mechanism.

Residue insertions that are not present in trypsin and chymotrypsin are found at the base of the most dynamic loops^3^, and are also sites of high local frustration^9^ suggesting evolutionary pressure for dynamic motions in these loops. The dynamic loops are concentrated on the side of the thrombin molecule furthest from the light chain, which clamps the linker between the N-terminal and C-terminal β-barrels and completely dampens μs-ms motions on the “back side” of thrombin. Thus, the double β-barrel architecture of thrombin, the light chain clamp, and the extended loops on the opposite face of the molecule allow thrombin to be a highly specific protease, while retaining dynamic malleability to respond to binding of its many allosteric effectors. It will be interesting to see if other serine proteases retain this same asymmetric distribution of dynamic motions.

Comparison of dynamics in apo-thrombin and PPACK-bound thrombin revealed that PPACK binding did not alter the ps-ns dynamics, but quenched most of the μs-ms motions that were present in the apo state. This result contrasts with the observation that in protein kinase A, inhibitor binding quenched both the ps-ns motions as well as the μs-ms motions^23^. Our results imply that a dynamic subset of thrombin conformers is selected from the broader ensemble of apo thrombin conformers upon PPACK binding and this subset retains residues undergoing μs-ms motions that form an allosteric pathway from the allosteric ABE1 site to the active site. Given the large number of different ligands that bind to thrombin, it is interesting to speculate whether specific functional activities of thrombin may retain subsets of dynamic motions selected from a broad and highly dynamic apo state as a result of specific binding of an allosteric effector. It will be interesting to see whether alternative motional pathways are selected for others of the many thrombin binding and catalytic functions.

ABE1 is a critical allosteric site. Fibrinogen must engage ABE1 in order to be efficiently cleaved at the active site, and thrombomodulin must engage ABE1 in order for protein C to be efficiently cleaved at the active site. Thermodynamic measurements showed that binding at ABE1 resulted in entropy-enthalpy compensation for ligands binding at the active site and vice-versa^24^. Such a thermodynamic compensation hinted that the sites may be coupled by dynamic allostery, however NMR was the only way to prove such coupling. The results presented here demonstrate that dynamic allostery is the information transfer mechanism between ABE1 and the active site of thrombin. Given that thrombomodulin binding to ABE1 is entropically-driven^40^, it will be very interesting to measure side chain dynamics in thrombin and to use these approaches to map the allosteric network more completely^41^,^42^,^43^.

## Methods

### Expression and purification of S195M thrombin

Details of the protein expression, refolding, and purification are as described previously^12^,^44^ with a few modifications. Due to the lack of auto-proteolytic activity of the S195M thrombin mutant, additional steps were required for activation of the mutant to the α-thrombin form. In brief, the S195M mutation was achieved by quick-change mutagenesis in pET23(+) containing the thrombin sequence with an 18 amino acid extension on the N-terminus (prethrombin-2(+18)) that is necessary for folding. [U-^2^H],[U-^15^N]- or [U-^2^H],[U-^15^N],[U-^13^C]-labeled S195M-thrombin was expressed in *E. coli* in minimal media containing the appropriate combinations of ^2^H_2_O, ^15^NH_4_Cl, and ^13^C-D-glucose. Protein inclusion bodies containing S195M-thrombin were isolated, and the protein was refolded and purified as previously described^12^. The protein is denatured in 6 M guanidine HCl and re-folded in H_2_O-buffer so all amides that may have been deuterated during growth are exchanged back for protons. Prethrombin-2(+18) was activated using 1 mg of *Echis carinatus* venom (Sigma-Aldrich) to cleave between the light and heavy chains and 700 μg of wild type α-thrombin (prepared previously and frozen at 4 °C in 350 μg/mL aliquots) was added to remove the N-terminal 18 amino acids of prothrombin. After activation (typically 18–24 hrs), the wild type α-thrombin was inhibited with biotinyl-PPACK (Haematologic Technologies) followed by addition of streptavidin resin (Thermo Scientific), and the biotinyl-PPACK-α-thrombin complex was removed via centrifugation. The α-S195M-thrombin was purified by MonoS ion exchange chromatography. Proper proteolytic activation, isotope incorporation, and removal of α-thrombin were confirmed by MALDI-TOF. Samples were buffer exchanged into NMR buffer: 25 mM sodium phosphate pH 6.5, 150 mM sodium chloride, and 0.05% sodium azide, with 10% v/v D_2_O added as a lock solvent. The final protein concentration in NMR samples was 0.15 mM.

### NMR resonance assignments and dynamics measurements

All NMR experiments were performed at 298 K on spectrometers equipped with cryogenic probes. Details of the experimental procedures for resonance assignments are outlined in Fuglestad *et al*.^12^. Experiments performed for resonance assignment were: HNCO, and HN(CO)CA at UCSD Pharmacology on a Bruker Avance III 600 MHz, TROSY-HN(CA)CO at NMRFAM on a Varian NMR system 600, TROSY-HN(CO)CACB and TROSY-HNCA at NMRFAM on a Varian VNS 800, and NOESY-^1^H,^15^N-TROSY with the UCSD Chemistry and Biochemistry Varian 800. Some assignments were transferred from the previously assigned PPACK-thrombin^12^ and additional assignments were made. Assignment transfers were confirmed with the 3D experimental data.

Forty residues for which N-H peaks were visible in PPACK-bound thrombin did not have visible peaks in the TROSY spectrum of apo-thrombin. These included E13(21) of the light chain; V17(38) which is adjacent to the N-terminus of the heavy chain; Q30(51) at the β-sheet leading to the 30 s loop; E61(92) of the 60 s loop; S83(115) and K87(119) of the strand connecting ABE1 and the 90 s loop; E97a-D100(130–133) of the 90 s loop and the catalytic aspartic acid, D102(135); T139-G140(175–176), G142(178), K145-A149A(181–186) and Q151(192) of the γ-loop; L160(201) and T177(218) on either side of the 170 s loop; C182(223) of a disulfide bridge; G188-E192(234–238) of the 180 s loop; G196(242) next to the catalytic serine; Y208-Q209(256–257); the active site adjacent W215(263) in the β-strand preceding the Na^+^-binding loop; C220-R221(267–269) of the Na^+^-binding loop; and Y225-G226(273–274), F232(280), and I238(286) of the C-terminus helix.

To calculate chemical shift differences, a weighted average approach was used to combine the differences in ^15^N and ^1^H chemical shifts, as described previously^45^. The average weighted chemical shift difference for each residue was calculated using the following equation:([(Δ*δ*_*HN*_)^2^+(Δ*δ*_*NH*_)^2^/25]/2)^1/2^. Residues with a weighted average chemical shift difference over 1 standard deviation (>0.11 ppm) were C42-A44(64–66) and T54(76) in between the 30 s and 60 s loops; the α-helix A56-C58(78–80) which includes the catalytic triad residue His57; L60(82) and K60f(88) of the 60 s loop; V66(97) at the base of the 70 s loop; Y89(121) in the β-strand connecting the 70 s and 90 s loops; R97(129) of the 90 s loop; R101(134) and I103(136) following the 90 s loop; I174(215) of the 170 s loop; A183-Y184a(224–226) at the base of the 180 s loop; V200(246) and M210(258) of the C-terminal β-barrel; S214(262), G216(264), and G219(266) of the Na^+^-binding loop; and F227-T228(275–276) and R233(281) near the base of the C-terminal helix.

NMR dynamics experiments for the calculation of order parameters (*R*_1_, *R*_2_, and ^15^N-{^1^H}NOE experiments) were performed at UCSD Pharmacology on a Bruker Avance III 600 MHz and analyzed as described previously^12^. For comparisons between the apo- and PPACK-bound thrombin, a consistent set of “rigid” residues was selected for the *R*_2_/*R*_1_ analysis using TENSOR2^46^ with a random snapshot from the MD simulation performed on apo-wild-type thrombin used as the structural model to fit the relaxation data to a rotational diffusion model. As with the previous analysis on PPACK-thrombin, no significant differences were observed for the isotropic vs. anisotropic diffusion model. The smaller set of residues selected for τ_c_ determination yielded a slightly larger τ_c_ than that previously reported^12^.

### R_2_ Relaxation Dispersion Experiments

The effective relaxation rates (*R*_2,eff_) due to contributions from conformational exchange on intermediate timescales were evaluated with Carr-Purcell-Meiboom-Gill (CPMG) experiments collected at NMRFAM on a Varian VNS 600 and a Varian VNS 800 using a TROSY-CPMG pulse sequence^47^. To ensure the observed line-broadening was not due to intermolecular associations line widths were measured at 0.06 and 0.1 mM with equivalent results. Based on these results, subsequent experiments were carried out at 0.1 mM. Two-dimensional data sets with 512 × 128 complex points or 2048 × 96 complex points, 32 scans were acquired at 1/τ_cp_ of 50, 100, 200, 300, 400, 600, 800, 1000, 1200, 1400, 1600, 1800, and 2000 Hz and a constant τ_CPMG_ = 40 ms with duplicates collected at 1/τ_cp_ of 50, 200, 1600, and 2000 Hz in order to estimate errors. For these experiments, Shigemi salt tolerant susceptibility matched slot NMR tubes were used. These tubes only require 170 uL of sample and need to be properly oriented inside the magnet to optimize S/N and minimize sample heating^48^.

The 600 MHz and 800 MHz *R*_2_ relaxation dispersion data for all apo-thrombin residues with *R*_ex_ > 6 s^−1^ (24 residues) were fit globally to the Richard-Carver equation^49^ by using the software package, GLOVE^30^. A cut-off of Rex > 6 Hz, was used which is equivalent to 2 times the maximal duplicate error. GLOVE minimizes global and local parameters alternately, and incorporates a Monte-Carlo minimization method to allow fitting parameters to pass through local minima. The following grid space was used in the Marquardt search: Δω = [100, 2500] (10 steps), *k*_ex_ = [5, 4000] (10 steps), p_B_ = [0.01, 0.1] (10 steps). The fit yielded the global exchange rate, *k*_ex_, the populations of the ground state (A) and excited state (B), where p_A_ + p_B_ = 1 and the residue-specific chemical shift difference between conformational states A and B (Δω). GLOVE fitting of the nine PPACK-thrombin residues with significant *R*_ex_ revealed fast exchange, and therefore the dispersion curves for all nine PPACK-thrombin residues were globally fit using the Meiboom equation^37^.

## Additional Information

**How to cite this article**: Handley, L. D. *et al*. NMR reveals a dynamic allosteric pathway in thrombin. *Sci. Rep.* **7**, 39575; doi: 10.1038/srep39575 (2017).

**Publisher's note:** Springer Nature remains neutral with regard to jurisdictional claims in published maps and institutional affiliations.

## Supplementary Material

Supplementary Information
