# Supplementary material for: NMR reveals a dynamic allosteric pathway in thrombin
Source: Sci Rep. 2017 Jan 6;7:39575. doi: 10.1038/srep39575 (PMC5216386; doi:10.1038/srep39575)
Supplement: Supplementary Information [file srep39575-s1.pdf]

Supplementary Figure for

NMR reveals a dynamic allosteric pathway in thrombin

Lindsey D. Handley[1,‡], Brian Fuglestad[1,3‡], Kyle Stearns[1], Marco Tonelli[2], R. Bryn Fenwick[4], Phineus R. L. Markwick and Elizabeth A. Komives[1*]

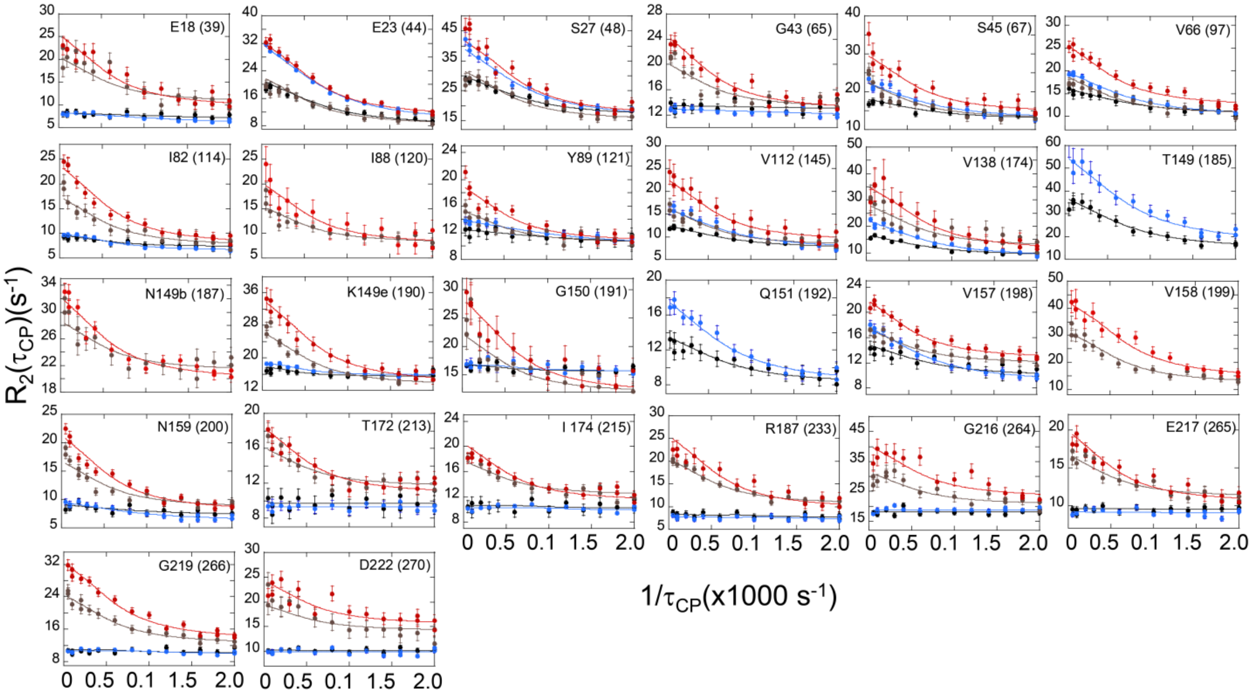

Supplementary Figure 1. Relaxation dispersion curves for apo-thrombin (800 MHz in red, 600 MHz in brown) and PPACK-thrombin (800 MHz in blue, 600 MHz in black). Curves are global fits from GLOVE.
